# Prospects of orphan crops in climate change

**DOI:** 10.1007/s00425-019-03129-y

**Published:** 2019-03-13

**Authors:** Tafadzwanashe Mabhaudhi, Vimbayi Grace Petrova Chimonyo, Sithabile Hlahla, Festo Massawe, Sean Mayes, Luxon Nhamo, Albert Thembinkosi Modi

**Affiliations:** 1 Centre for Transformative Agricultural and Food Systems, School of Agricultural, Earth and Environmental Sciences, University of KwaZulu-Natal, P/Bag X01, Scottsville, Pietermaritzburg, 3209 South Africa; 2 School of Biosciences, University of Nottingham Malaysia, Jalan Broga, Seminyih, Selangor Darul Ehsan Malaysia; 3 Crops for the Future, Jalan Broga, Semenyih, Selangor Darul Ehsan Malaysia; 4 International Water Management Institute, Southern Africa (IWMI-SA), 141 Cresswell Street, Silverton, Pretoria, 0184 South Africa; 5 School of Biosciences, University of Nottingham, Sutton Bonington Campus, Leicestershire, LE12 5RD UK; 6 Department of Plant, Soil and Microbial Sciences, Michigan State University, 1066 Bogue St., Rm. A286, East Lansing, MI 48824 USA

**Keywords:** Adaptation, Food and nutrition security, Genetic diversity, Resilience, Sustainability

## Abstract

Orphan crops can contribute to building resilience of marginal cropping systems as a climate chnage adaptation strategy.

Orphan crops play an important role in global food and nutrition security, and may have potential to contribute to sustainable food systems under climate change. Owing to reports of their potential under water scarcity, there is an argument to promote them to sustainably address challenges such as increasing drought and water scarcity, food and nutrition insecurity, environmental degradation, and employment creation under climate change. We conducted a scoping review using online databases to identify the prospects of orphan crops to contribute to (1) sustainable and healthy food systems, (2) genetic resources for future crop improvement, and (3) improving agricultural sustainability under climate change. The review found that, as a product of generations of landrace agriculture, several orphan crops are nutritious, resilient, and adapted to niche marginal agricultural environments. Including such orphan crops in the existing monocultural cropping systems could support more sustainable, nutritious, and diverse food systems in marginalised agricultural environments. Orphan crops also represent a broad gene pool for future crop improvement. The reduction in arable land due to climate change offers opportunities to expand the area under their production. Their suitability to marginal niche and low-input environments offers opportunities for low greenhouse gas (GHG) emissions from an agro-ecosystems, production, and processing perspective. This, together with their status as a sub-set of agro-biodiversity, offers opportunities to address socio-economic and environmental challenges under climate change. With research and development, and policy to support them, orphan crops could play an important role in climate-change adaptation, especially in the global south.

## Introduction

Climate change is one of the global challenges facing mankind today as temperatures continue rising, triggering a host of extreme weather events such as heat waves, drought, and flooding (Feulner 2017). These climate-induced challenges are manifesting themselves rapidly, causing socio-economic insecurities and health challenges, particularly in marginalised communities (Schmidhuber and Tubiello 2007). There is increasing evidence of indirect associations between climate change and the rise in the rates of malnutrition, poor health, hunger and starvation, as well as food and water insecurity (Bain et al. 2013; Padulosi et al. 2013; Wheeler and von Braun 2013). In addition, climate-change impacts have put an additional pressure on already stressed natural resource base, reducing the resilience of agro-ecosystems that are, in part, providing food and nutritional security in rural communities (Naluwairo 2011). Tackling these challenges requires a paradigm shift from the current incremental adaptation strategies towards transformative alternatives that also place an equal emphasis on human nutrition and health, as well as environmental sustainability (Francis et al. 2017). In the context of marginalised farming communities, a transformative adaptation strategy is defined as one that causes a disruptive, but desirable and sustainable change to the social–ecological state of the system (Lonsdale et al. 2015). In the context of this paper, the inclusion of adaptable nutrient dense orphan crops into marginalised agricultural systems and dominant food systems is considered part of transformative adaptation (Mabhaudhi et al. 2019).

Orphan crops are defined as crops that have either originated in a geographic location or those that have become ‘indigenized’ over many years (> 10 decades) of cultivation as well as natural and farmer selection (Dawson et al. 2007). The term ‘orphan’ has often been used to refer to crops that may have originated elsewhere, but have undergone extensive domestication locally, thus giving rise to local variations, i.e., ‘naturalized/indigenized crops’ (Mabhaudhi et al. 2017a). Indigenized crops are sometimes referred to as orphan crops (Mabhaudhi et al. 2017a). Underutilised indigenous and traditional crops are often characterized by the limited development relative to their potential. Consequently, they have poorly developed and understood value chains and these vary across geographic and socio-economic settings. Several research findings have advocated for their use as a part of sustainable agriculture techniques that speak to adaptation, mitigation, and sustainable intensification of production systems (Branca et al. 2011; Grainger-Jones 2011; Tilman et al. 2011).

There is growing recognition that the use of locally available resources such as orphan crops can contribute to adapting to climate variability and change (Bvenura and Afolayan 2015; Dawson and Jaenicke 2006; Padulosi et al. 2002a) while supporting sustainable diets and food systems. Orphan crops may offer ‘new’ opportunities in the advent of climate change as they are uniquely suited to local harsh environments, provide nutritional diversity, and enhance agro-biodiversity within farmer fields and home gardens (Table 1), create niche markets in local economies, and serve to simultaneously harness and protect local knowledge (Massawe et al. 2015). Furthermore, they are a mainstay of rural food systems. However, these reported benefits are largely anecdotal with limited empirical evidence. When compared to major crops, research has also shown that most orphan crops are low yielding and have limited benchmarking resulting in low adoption in mainstream farming systems (Chivenge et al. 2015; Mabhaudhi 2009; Mayes et al. 2012). Despite the inherent low-yield potential exhibited by orphan crops, the fact that they have persevered with a little formal support suggests they may be resilient and possess certain desirable traits within communities who utilise them which may be useful for climate-change adaptation. In this regard, orphan crops may contribute to building resilience of the communities over the long-term.

**Table 1:** Examples of underutilised crop diversity observed across different land units

Land unit	Main crop species	Underutilised crops
Communal croplands^1^ and out fields	Maize (*Zea mays* L.), Dry bean (*Phaseolus vulgaris*)	Sorghum (*Sorghum bicolor*), millets, African eggplant (*Solanum macrocarpon*, *S. aethiopicus* and *S. anguivi*), vegetable and grain cowpea (*Vigna unguiculata*), wild mustard (*Brassica carinata*) and jute mallow (*Corchorus olitorius*) Chinese cabbage (*Brassica rapa* L. subsp. Chinensis), pumpkin (*Cucurbita maxima*), various *Cucurbita* spp., bitter watermelon (*Citrullus lanatus* (Thunberg) Matsum. subsp. Lanatus), spider plant (*Cleome gynandra* L.) cassava (*Manihot esculenta*), sweet potato (*Ipomoea batatas*), bambara groundnut (*Vigna subterranea*) and taro (*Colocasia esculenta*), Fat hen (*Chenopodium album*), Spindle-pod (*Cleome monophylla*), Jelly melon (*Cucumis metuliferus*), Devil’s thorn (*Tribulus terrestris*), Gallant soldier (*Galinsoga parviflora*), Yellow justicia (*Justicia flava*), Stars talk (*Oxygonum sinuatum*), Sticky gooseberry (*Physalis viscose*), Purslane (*Portulaca oleracea*), Coffee senna (*Senna occidentalis*), Black nightshade (*Solanum nodiflorum*) and Giant bell flower (*Wahlenbergia undulata*)
Home gardens^2^	Pumpkin, wild mustard, tomatoes, onions, spinach, cabbage	Maize landraces, sorghum, millets, African eggplant, vegetable and grain cowpea, jute mallow, Chinese cabbage, various *Cucurbita* spp., bitter watermelon, spider plant, sweet potato, bambara groundnut, Fat hen, Spindle-pod, Jelly melon, Devil’s thorn, Gallant soldier, Yellow justicia, Stars talk, Sticky gooseberry, Purslane, Coffee senna, Black nightshade, and Giant bell flower

^1^Communal croplands are fields often located in rural and in belong to a community, rather than an individual or company, and administered by customary law. Individuals belong to the community can either lease or get allocated (Van Averbeke and Khosa 2007)

^2^Homestead gardens, also known as home gardens, are small-scale production system supplying food and medicinal products either not obtainable, affordable, or readily available through retail markets, field cultivation, hunting, gathering, fishing, and wage earning (Van Averbeke and Khosa 2007)

## Climate change and food systems

The drivers of climate variability and change are well reviewed (de Groot et al. 2006; Travis 2013; Vermeulen et al. 2012). In the short term, increasing climate variability has a greater impact on food security than longer term changes in mean climate values (Preston 2014). Agriculture needs to adapt to sporadic and gradual changes in means and distributions of temperature and precipitation (Nelson et al. 2010). Depending on the speed and direction of these trends, adaptation needs to be reconceptualized as an unabating and transformative process, rather than intermittent and incremental (Lonsdale et al. 2015). Under continuously changing conditions, transformative adaptation is needed to build resilience and ensure sustainable food systems (Wheeler and von Braun 2013).

Food systems include all processes and infrastructure involved in feeding a population, namely, growing, harvesting, processing, packaging, transporting, marketing, consumption, and disposal of food and food-related items (Preston 2014). Theoretically, the supply and demand of food should balance global food systems; however, the imbalances in the drivers have caused food systems to be in flux (Myers et al. 2017). Population growth, shifting consumption patterns, urbanization, and income re-distribution have driven the demand side (Myers et al. 2017; Wheeler and von Braun 2013). Patterns in food supply-side drivers, which are related to resource use (water, energy, and land), and agro-ecosystem services have not always been able to provide for the growing demand (Wheeler and von Braun 2013). In addition, the impacts of global climate change on food systems are complex as they are widespread, they differ across spatial and temporal scales, and are influenced by pre-existing and emerging governance, and social and economic conditions (Meybeck and Gitz 2016; Pelletier et al. 2011; Wheeler and von Braun 2013).

There is sufficient evidence that shows that climate change will affect crop yields and quality (Shindell et al. 2012). A change in the observed climate will affect the growth of crops through multiple mechanisms, including changing phenology, heat stress, water stress, waterlogging, and increases or reductions in pests and diseases (Venkateswarlu and Shanker 2009). Based on general circulation models, the forecasted yield changes in 2050 are estimated to be between − 27 and + 9% across all the developing countries for the three key staple crops [maize (*Zea mays* L.), rice (*Oryza sativa*), and wheat (*Triticum aestivum* L.)], assuming a carbon fertilization effect (Nelson et al. 2010; Zargar et al. 2017). According to Scheelbeek et al. (2018), the impacts of environmental changes on nutritional quality remain unclear for vegetable/legume and fruit crops due to scanty quantitative reports. On the other hand, there are overwhelming data that show clear impacts in cereal crops (Beleggia et al. 2018; Chaturvedi et al. 2017; Giri et al. 2016; Manderscheid et al. 1995; Scheelbeek et al. 2018; Smith and Myers 2018). Higher carbon dioxide concentration, for example, is shown to lower concentrations of zinc, iron, and protein and raise starch and sugar content in crop plants that use three-carbon fixation pathway such as wheat, rice, and soybeans (*Glycine max*) (Vermeulen et al. 2012). However, to our knowledge, there are no published reports that show the impact of climate change on nutritional quality in orphan crops. Anecdotal reports often suggest that, due to the generations of farmer and natural selection, nutritional quality of orphan crops may be resilient to climate shocks. However, similar to the other anecdotal reports of orphan crops’ potential, this requires empirical evidence to be confirmed.

This review evaluates the role that orphan crops could potentially play under climate change, especially in marginal production areas. More specifically, the review seeks to identify the potential of orphan crops to contribute to (1) sustainable and healthy food systems, (2) genetic resources for future crop improvement, and (3) agricultural sustainability under climate change. Finally, the review addresses (4) co-benefits of including orphan crops under climate change.

## Methodology

A mixed method review approach, which included combining quantitative and qualitative research or outcomes with the process studies, was used to compile the review. Articles were identified through searches on Google Scholar databases for the period from 1995 to December 2018. Other search engines, Scopus, ScienceDirect, and SpringerLink, were also used. The primary search terms used were “climate change”, “drought”, “extreme weather”, “traditional”, “indigenous”, “neglected”, “orphan”, “new”, “future”, or “neglected and underutilised crop species”. The search did not discriminate by searching within keywords, titles, and abstracts, but searched for key terms throughout the articles. As such, the search was largely web-based and designed to cover both grey and academic literature. The main advantage of this was that it extended the search beyond articles that would normally be unavailable for the audience outside research. The number of hits was ~ 5000 and these were screened for relevance to the objectives of the review;   ~ 109 were then cited in the text.

The first section of the review explores the prospects of orphan crops under climate change by focussing across several bio-physical and socio-economic domains. Concepts of sustainable food systems, agro-ecosystem function and services, land reclamation, and plant breeding and genetic manipulation are discussed in relation to orphan crops. The objective was to highlight the attributes of orphan crops that best exemplify their potential to deliver on building resilient, diverse, and sustainable global diets. The second section details the co-benefits of orphan crops with respect to health, environment, and socio-economic challenges. Climate change is a complex problem which cuts across sectors. Responses to climate change will also require solutions that offer cross-cutting opportunities in terms of adaptation, mitigation, and sustainability. This section, therefore, explores the possible co-benefits of orphan crops to these sectors. The third and final section then discusses several limitations that would need to be addressed if the potential of orphan crops was to be realised under climate change.

## Results and discussion

### Sustainable and healthy food systems

A sustainable food system delivers food and nutrition security for all in a way that is economically, socially, and environmentally sound so as not to compromise food security and nutrition for future generations (Van Esterik 2005). Sustainable food systems also allow for sustainable diets which have been defined as diets with low environmental impacts which contribute to food and nutrition security and to healthy life for the present and future generations (FAO 2010). Malnutrition results from deficiencies, excesses, or imbalances in the consumption of macro- and/or micronutrients (Ravi et al. 2010), and it includes both under- and over-nutrition (Faber et al. 2011). To date, 821 million people, mostly in South Asia and Africa, are estimated to be undernourished and lack enough food and, another 2 billion people are overweight (FAO et al. 2018). Furthermore, 151 million children under the age of 5 suffer from some form of malnutrition (under- or over-nutrition), which accounts to 45% of deaths in children in the same age group (Baldermann et al. 2016; Black et al. 2013). There is need to invest in nutrition-sensitive agricultural strategies that provide sustainable diets and can continuously contribute to addressing malnutrition challenges under changing climate.

Currently, total demand for cereals in sub-Saharan Africa is projected to increase from 2.26 to 2.60% per year during 2025–2050 (Ringler et al. 2010). Net cereal imports into sub-Saharan Africa are also expected to increase by 2050 (van Ittersum et al. 2016). For example, rice imports are expected to rise by 57.8% from 2000 to 2050 under climate change (Ringler et al. 2010). Instead of relying heavily on the importation of food, a cost-effective strategy to diversifying crop production to meet both food and nutrition needs of a growing population could be that of exploiting climate resilient and nutritious orphan crop species (Kumar and Reinartz 2018; Massawe et al. 2016) and mainstreaming them into the existing food systems (Mabhaudhi et al. 2019). For example, millets and gluten-free grains, such as amaranth (*Amaranthus caudatus*), teff (*Eragrostis tef*), and quinoa (*Chenopodium quinoa*) are rich in vitamins, minerals, essential fatty acids, phyto-chemicals, and antioxidants (Kumar and Reinartz 2018), and are considered to be more drought tolerant than their commercial counterparts. Grains from these species have been trending in many western countries as an ideal food source, being gluten free with balanced protein essential for human health (Massawe et al. 2016). While there is emerging evidence to show the low levels of nutrients such as protein, zinc, and iron for major staple crops grown under elevated CO_2_, there are no similar studies for orphan crops (Gangi et al. 2015; White et al. 2011). Thus, there is an urgent need to conduct comparative studies to determine the effect of climate change (e.g., elevated CO_2_, drought, and high temperature) on nutritional quality of orphan crops. This would contribute to confirming anecdotal reports of orphan crops’ potential.

Several orphan crops are nutrient dense and their ability to adapt to harsh conditions suggests that they can be deployed as part of efforts to champion climate-change adaptation, agriculture, and economic advancement for smallholder farmers residing in low-production environments (Padulosi et al. 2011). Orphan crops already form part of local food systems, suggesting that they are socially acceptable (Rojas et al. 2009). Seeds of orphan crops are often locally sourced, and their production systems do not require large amounts of fertiliser and other agrochemicals making them a cost-effective and environmentally friendly option for resource poor communities (Dawson et al. 2007). In addition, their vast genetic pool presents an important sub-set of agro-biodiversity; therefore, they strengthen and enhance farm-level resilience to perturbations (Padulosi et al. 2011). There are also strong linkages between high agro-biodiversity and the ability of food systems to deliver adequate quantities of healthy and nutritionally balanced food (Gillespie and van den Bold 2017; Rosin et al. 2011). In this regard, orphan crops could support and strengthen the existing food systems to deliver sustainable diets as they are economically, socially, and environmentally sound, and offer cross-cutting solutions (Mabhaudhi et al. 2017b). Incidentally, the continued existence of orphan crops within marginalised farming systems shows that they are adaptable to changing climates making them front runners for transformative adaptation.

The fact that orphan crops’ value chains are currently poorly developed creates opportunities to further diversify current food systems, thus creating new employment, improving access to nutritious foods, increasing market and distribution opportunities, and promoting autonomous pathways out of poverty (Mabhaudhi et al. 2017b). Importantly, the significant role played by women in the production and conservation of orphan crops offers opportunities for gender empowerment through their involvement in the food systems (Mabhaudhi et al. 2017a, 2019). Promoting gender equality and women empowerment is inextricably linked to the strengthening of sustainable food systems to fight hunger and malnutrition and improving the livelihoods of rural populations (Ngcoya and Kumarakulasingam 2017).

Including orphan crops as part of transformative adaptation allows for adapting to the climatic, ecological, and natural limits in which resource poor farmers, in particular, reside (Pérez-Català 2014). Orphan crops can, therefore, offer opportunities for ‘fitting to’ or ‘fitting with’ the socio-ecological environment while trying to sustainably maintain the natural products or processes that are needed for sustainable food systems (Chivenge et al. 2015). In terms of ‘fitting to’, orphan crops can contribute to transformational adaptation through (1) upscaling adaptation efforts, (2) introducing new forms of adaptation strategies to a new region, and (3) holistic transformation of behaviour and place—landscape transformation (Mabhaudhi et al. 2017a). In addition to this, orphan crops can drive co-evolution of food systems, ‘fitting with’ the same socio-ecological environment rather than seeing it as something external (Mabhaudhi et al. 2019).

## Genetic resources for future crop improvement

In general, orphan crops are grown at small scale, often as mixed genetic populations rather than pure lines, with minimal inputs and on marginal land (Mabhaudhi et al. 2016). While selection of the landrace to the local environment and agricultural system has happened through the constant cultivation of the crop in location, intensive selection for high input and uniform agricultural systems has not (Massawe et al. 2016; Mayes et al. 2012). Several research findings have shown that trait variation present within a crop species is a major determinant of what can be achieved through breeding; to combine the beneficial gene alleles into a single ‘ideotype’ (Donald 1968; Gutschick 1987). Most orphan crops still contain gene alleles and mechanisms for growth in poor environments and for resilience under stress (Voegel et al. 2012). These have potentially been lost from major crops (and, hence, programmes to introduce variation from wild ancestors for major crops) (Ellstrand et al. 2010). However, there are many disadvantages with assessing the genetic potential of orphan crops, not least, which ones to focus on. If we at least select species with beneficial traits outside the range seen in major crops (whether this is drought tolerance, nutritional concentration or other aspects which are useful for healthy diets), then they will be a sub-set of crops which could have a major impact on food and nutritional security (Massawe et al. 2016; Mayes et al. 2012).

Until recently, we knew little about the breeding systems and pollination mechanisms in many crops (and particularly for tree species) or the genetic relatedness of accessions within a species. The development of Next-Generation Sequencing (Goodwin et al. 2016) and particularly generic genotyping approaches such as Genotyping-by-Sequencing (https://www.diversityarrays.com/ for one approach) have permitted a step change in our ability to interrogate these systems. The only prerequisite for genetic analysis by GBS is the ability to extract DNA which can be reliably cut by bacterial endonucleases (Goodwin et al. 2016). By collecting trees or crop plants within a population and multiple seed from individuals, we can begin to get a clearer idea of the breeding systems and the factors (insect, distance, prevailing wind, etc.) involved in pollination.

An objective for many inbreeding major crop species is to develop hybrids. While development of hybrids would offer potential yield increases and greater hybrid vigour (Elhani et al. 2007; Mabhaudhi 2009), it is unlikely to be achieved for years in many orphan crops. However, for orphan crops, there has often been relatively little conventional breeding and a significant progress could be made by a well-organised conventional breeding programme (Padulosi et al. 2002b). As more information is generated at the genetic and trait levels from orphan crop species, it becomes more feasible to include marker-assisted selection as an integrated component of the breeding programme. The development of the African Orphan Crops Consortium (AOCC; http://www.africanorphancrops.org), including a remit to generate the genome sequence of 101 African Orphan Crop Genomes and re-sequence 100 lines of each, should accelerate the adoption of molecular breeding and research in these crops. The first five genome assemblies have been released for bambara groundnut (*Vigna subterranea)*, lablab (*Lablab purpureus*), white acacia (*Faidherbia albida*), marula (*Sclerocarya birrea*), and moringa (*Moringa oleifera*) (Chang et al. 2018), and hopefully represent the beginning of a new genome-enabled breeding era for orphan crops.

The release of the genome sequences will be important, but to be able to exploit the new information, there is a need to develop structured genetic material for both research and breeding to elucidate the genetic control of traits and develop markers. Such material also could allow selections by farmers in the target location (which requires suitable adaptation and farmer preference traits, fitting into the current agricultural systems). For orphan crop systems, we do not have the extensive history of pedigree-based approaches which exists for more major species and often collections of orphan crop accessions will have very distant divergence times. This potentially makes the use of Nested Association Mapping (NAM) (Buckler et al. 2009; Yu et al. 2008), Multiparent advanced generation intercross populations (MAGIC) (Bandillo et al. 2013; Gardner et al. 2016; Huang et al. 2015), and even Genomic Selection (GS) (Jannink et al. 2010) and examples from wheat and oil palm (*Elaeis guineensis*) (Bassi et al. 2016; Kwong et al. 2017) of interest. Such populations could be deployed in many locations, with local farmers identifying the material most suited to their needs and preferences.

The challenges presented by climate change are significant and probably the greatest test of the ingenuity of humankind. Expanding our range of crops through application of genetic and genomic tools—including de novo domestication—is one important route to sustainable food and nutritional security and is also achievable. In addition, the broadening of crop choices within agricultural systems will contribute to transformative adaptation efforts.

### Agricultural sustainability under climate change

#### Land use

Of the 83 million ha of arable land in southern Africa, 9 million is irrigated and 74 million is rainfed. Approximately 89% of this land is used to produce major crops under rainfed agriculture, but still failing to meet the food requirements of the region as about US$35 billion is spent on food imports to supplement food deficits (FAO 2003). As climate change threatens to reduce the land suitable for production of major crops, this could inevitably open up more land for orphan crops that do well under extreme climate and edaphic conditions (Carter and Gulati 2014). Crops such as finger millet, cowpea, and bambara groundnut are adapted to extreme weather (drought and heat stress) and poor soil conditions (Tadele 2017). Due to their inherent tolerance to water deficit, they are cultivated under semi-arid conditions (Chivenge et al. 2015). Research has shown that several orphan crops require less water and have relatively high water use efficiencies (Chibarabada et al. 2017; Hadebe et al. 2017) (Table 2). They can also be grown in marginal and fragile environments, such as dry lands and swamps, and on highly degraded land that is no longer suitable for high input commercial crops (Baldermann et al. 2016; Naluwairo 2011). Therefore, land that has been condemned as unsuitable for cultivation of major crops may be suitable for cultivating adaptable orphan crops (Mabhaudhi et al. 2017b). However, the cultivation and expansion of orphan crops at a large scale must be supported with crop suitability mapping for effective matching of specific orphan crops to suitable climates (Mabhaudhi et al. 2017b).

**Table 2:** Water use and water use efficiency (WUE) of selected underutilised cereal and legume food crops. Table adapted from Chibarabada et al. (2017) and Hadebe et al. (2017)

Crop species	Water use	WUE (kg ha^−1^ mm^−1^)
Groundnut (*Arachis hypogaea*)	697–809	3.96–5.25
Bambara groundnut (*Vigna subterranea*)	582–856	0.09–0.11
Chickpea (*Cicer arietinum*)	150–340	1.90–3.60
Cowpea (*Vigna unguiculata*)	78–258	0.11–0.20
Faba bean (*Vicia faba*)	101–261	1.7–12.5
White lupine (*Lupinus albus*)	178–272	21–8.5
Sorghum (*Sorghum bicolor* L.)	450–650	12.4–13.4
Finger Millet (*Eleusine coracana*)	450–650	5.1–10.4
Tef (*Eragrostis tef*)	450–550	4.2–11.2
Maize (*Zea mays* L.)	500–800	3.2–5.7

Crop suitability mapping is an assessment of land performance when it is used to produce specific crops (Li et al. 2009). Adaptation of crop growth to the capabilities and constraints of local agro-ecological conditions is a key principle of sustainable land management (Bharucha and Pretty 2010; Pretty and Bharucha 2014) and for climate-change adaptation. Identifying optimum land for cultivation of orphan crops is necessary for the conservation of environmental resources and, at the same time, achieving optimum yields (Eastman 1999). In addition, identifying optimum land suitable for cultivating orphan crops is essential for producing more with fewer resources for sustainability. Thus, cropland suitability mapping provides information for growing potential crops and deriving maximum economic benefits with lower production costs (Kihoro et al. 2013). Crop suitability mapping will facilitate a better utilisation of marginal land and water resources, providing opportunities to produce orphan crops in areas that are projected to become unsuitable for the production of major crops. This is consistent with transformational adaptation which has also been defined as fitting to or fitting with the socio-ecological landscapes, depending on how it is conceptualized.

#### Enhancing ecosystem goods and services

There is limited literature on the role played by orphan crops with regards to provision of ecosystem services. While there is growing recognition in the literature highlighting their potential, especially in sub-optimal environments and under climate change, there is scant literature describing ecosystem services which they provide. What has been established is that orphan crops can deliver provisioning and cultural ecosystem services and this hinges on the biodiversity that exists within them. There are over 12,000 crop species worldwide that are classified as suitable for human consumption, yet the world is fed by only 30 crops (Adhikari et al. 2017). Among this select group, rice, maize, and wheat provide approximately 60% of the world population’s dietary energy (Shiferaw et al. 2011). This is mainly due to strong policy support, targeted breeding efforts that have made them highly adaptable to several environments, high calorific value, and product versatility (Ebert 2014). Orphan crops form part of a species rich sub-set of agro-biodiversity.

The cultivation of orphan crops suited for local environments could provide nutritional diversity for communities, and an option for crop rotation for farmers, creates niche markets in local economies, and harnesses and protects local knowledge (Padulosi et al. 2013) and agro-biodiversity (Gaisberger et al. 2016). Orphan crops provide opportunities for farmers to disrupt pest and disease cycles, replenish nutrients through improved contributions and support of nutrient cycling, and increase the presence of pollinators (Chapin III et al. 2000). Orphan crops can, therefore, be considered as protective and respectful of biodiversity and ecosystems (FAO 2010). Thus, harnessing local knowledge and the use of orphan crop species has enormous potential to improve food security in the developing countries under climate change (Vorster 2007). With the risk of a shrinking food basket under climate change, mainstreaming orphan crops into local food systems will mitigate malnutrition, which is predicted to also increase under climate change. However, despite this reported potential, orphan crops still face significant obstacles with regards to being mainstreamed into the dominant agricultural landscapes and food systems.

### Benefits of including orphan crops in climate-change agenda

#### Climate-health co-benefits of orphan crops

Millions of people in the global South rely on orphan crops as primary food sources. Numerous studies have shown that these crops are highly nutritious, containing several micro- and macro-nutrients that are essential for health, more so than common major crops (Kour et al. 2013; Magbagbeola et al. 2010; Nyadanu and Lowor 2015; Tadele 2018). For example, several traditional cereals, legumes, and vegetable crop species, in particular, contain high proportions of vitamins, calcium, iron, potassium, magnesium, and zinc, and some orphan fruits and vegetables contain more vitamin C and pro-vitamin A than major crop species and their staple counterparts such as maize (Kour et al. 2018; Tadele 2018) (Table 3). Certain orphan crops also have been reported to have certain health protection and medicinal properties, and can have protective effects against the major chronic diseases. For example, finger millet has a low glycemic index and can be digested slowly, making it popular among diabetic patients (Tadele 2018). Finger and pearl millets have anticancer properties and might have potential to contribute to the prevention of cancer initiation due to the phenolic extracts which they contain (Tadele 2018). Depending on species, the inclusion of orphan crops into low-income household diets can improve the availability of some essential nutrients, especially essential amino acids, fibre, proteins, and promote dietary diversity. This makes them an important component for nutritious diets (Nyadanu and Lowor 2015) and should be part of a basket that still includes major staple crops with high nutritional value.

**Table 3:** A comparison of the nutritional value (based on raw 100 g portion) of selected orphan crops. Adapted from Mabhaudhi et al. (2017a)

	Common name	Energy (kcal)	Protein (g)	Fat (g)	Fibre (g)	Ash	CHO (g)	Ca (mg)	P (mg)	Na (mg)	Mg (mg)	Cu (mg)	Zn (mg)	Fe (mg)
Cereals	Maize (*Zea mays* L.)	339	13.7	2.47	2.7	1.78	71	34	508	2	3.01	0.55	4.16	3.01
	Sorghum (*Sorghum bicolor* L.)	329	10.9	3.2	2.3	1.6	73	27	215	4	103	0.3	1.5	2.6
	Finger millet (*Eleusine coracana*)	363	11	5	2.2	1.9	69	25	–	–	–	–	–	–
	Pearl millet (*Pennisetum glaucum*)	378	9.0	4	1.5	1.9	84	23	10					1.8
	Tef (*Eragrostis tef*)	367	13	2.4	8	2.49	73	0.19	13	0.01	354.18	–	37.30	50.78
Legumes	Bambara (*Vigna subterranea*)	386.32	21.85	6.9	3.42	3.6	53.39	219.26	266.1	11.9	2.6	0.41	7.9	7.02
	Cowpea (*Vigna unguiculata* (L.) Walp.)	357.1	24.7	4.8	2.8	4.2	51.76	180.46	310.9	107.24	1.74	9.9	5.3	4.9
	Dry bean (*Phaseolus vulgaris*)	333	21.8	2.5	1.8	4.1	2.5	183	–	101	–	–	–	4.7
	Lablab (*Lablab purpureus*)	117	26.86	0.27		3.96	67.23	–	8	–	–	–	0.38	0.76
	Sword Bean (*Canavalia gladiate*)	1560.3	28.39	7.84	8.23	5.63	49.91	–	–	–	–	–	–	–
	Marama bean (*Tylosema esculentum*)	477	34.71	40.06	3.94	3.19	14.07	241	454	63.75	274.5	1.04	6.2	3.95
Root and tuber crops	Taro (*Colocasia esculenta*)	102	7.79	0.65	3.01	2.44	86.11	55	1.6	–	–	–	1.67	–
	Sweet potato (*Ipomoea batatas*)	86	1.6	0.1	3.0	1.05	20.1	30	47	55	25	3	249	0.42
	Cocoyam (*Colocasia esculenta*)	112	1.5	0.2	4.1	–	26	–	–	–	–	–	–	–
Vegetables	Amaranth (*Amaranthus* spp)	49	4	0.2	2.87	3.42	7.86	1686	487	347	82	3	56	25
	Nightshade (*Solanaceae* spp)	55	3	0.6	2.42	2.24	9.03	2067	478	431	3	6	23	85
	Black jack (*Bidens pilosa*)	39	5	0.6	2.92	2.82	3.72	1354	504	290	21	10	22	17
	Jews Mallow (*Corchorus olitorius*)	392	20.90	5.20	45.61	–	55.50	1760	490	801.20	15.50	11.30	12.40	53.30
	Wild mustard (*Sinapis arvensis*)	26	2.7	0.2	1.1	1.4	4.9	–	–	–	–	–	–	–
	Bottle gourd (*Lagenaria siceraria*)	14	0.62	0.02	0.5	0.5	3.39	26	13	2	0.089	0.034	0.70	0.20
	Chinese Cabbage (*Brassica rapa* subsp. Pekinensis)	21	9	1	1.0	1.4	22	152	32	29	42	0.07	0.30	1.4
	Sun–berry (*Solanum retroflexum*)	38	5.8	0.8	1.4	8.8	5.0	442	75	–	–	–	–	4.2
	Wild water melon (*Citrullus Lanatus* L.)	296	3.5	0.4	3.8	1.66	13.1	212	119	9	59	0.20	0.74	6.4

Orphan crops can offer new opportunities to address malnutrition and food insecurity, which are exacerbated by the rapidly increasing global population, the reduction in arable land, and the changing climate. In this regard, orphan crops offer opportunities to co-evolve, hence transform socio-ecological landscapes, in response to changing socio-economic and bio-physical factors and the need for healthier diets. Changes in the traditional food habits have resulted in an overdependence on energy-rich, and nutrient poor staple crops, especially in more affluent households where there is a high consumption of red and processed meat, dairy products, and eggs, and a relatively low consumption of fruits and vegetables (Nyadanu et al. 2015; Springmann et al. 2016a). Therefore, augmenting meat with more plant-based products from orphan crops which contain some of the same nutrients, could also lead to sustainable and healthier diets, and reduce the impact of the global food system on the environment. Research shows that worldwide adoption of a more plant-based diet could contribute to the reduction of food-related greenhouse gas emissions by up to 70% by 2050 (Springmann et al. 2016a).

While orphan crops may contain high levels of certain nutrients, they also contain low levels of certain nutrients and, in some cases, anti-nutritional factors. These anti-nutritional factors make them less palatable and difficult to process (Chibarabada et al. 2017; Chivenge et al. 2015; Dawson et al. 2007). For instance, alpha-galactosides, the main flatus-causing compounds are present in red and white lima beans (*Phaseolus lunatus*), brown and cream pigeon pea (*Cajanus cajan*), African yam bean (*Sphenostylis stenocarpa Hochst* ex A. Rich), bambara groundnut, and jack bean (*Canavalia ensiformis*) (Oboh et al. 1998). These challenges, among others, could be overcome through concerted crop improvement programmes for orphan crops, but they currently limit efforts to exploit the full value of orphan crops for climate-change adaptation.

#### Climate–environment co-benefits of orphan crops

Orphan crops are important for the conservation of agricultural biodiversity and agro-ecosystems which are critical for the long-term sustainability of food and agricultural production (Baldermann et al. 2016; Naluwairo 2011). In addition, the adoption of orphan crops could contribute towards the reduction of greenhouse gas (GHG) emissions (Chivenge et al. 2015). Food systems, through the conversion of natural lands to agricultural land for crop and/or livestock production, and the intensification of production on the existing agricultural lands, make up approximately 19–30% of global anthropogenic GHG emissions (Donati et al. 2016; Hallström et al. 2015; Springmann et al. 2016b). The production of food from animals utilises large areas of land and, as a result, bears greater environmental impacts than fruit and vegetable production due to the high levels of nitrogen and GHG emissions (Donati et al. 2016; Stehfest et al. 2009). Springmann et al. (2016a, b) report that approximately 80% of the emissions from food systems are associated with livestock production. Hence, our dietary food choices affect our health and the environment. Orphan crops can also reduce the contribution of environmental contaminants by agriculture. Orphan crops can tolerate pests and diseases, and grow in soils of low quality and are known to require lower levels of inputs such as pesticides and fertilizers.

#### Climate-socio-economic co-benefits of orphan crops

Orphan crops can provide and improve income for the poor, especially women and youth, who generate income from agricultural activities, particularly in rural areas (Kour et al. 2018; Naluwairo 2011). As previously alluded (cf. Climate–environment co-benefits of orphan crops), orphan crops require low levels of inputs such as pesticides and fertilizers, which reduces input costs for farmers (Dansi et al. 2012; Kour et al. 2018). They are also resistant to pests and diseases, and tolerant to environmental extremes and less favourable weather conditions, unlike major crops (Chivenge et al. 2015; Naluwairo 2011; Tadele 2018), meaning that the source of income for the farmers will not be disrupted. Within communities, orphan crops can offer cross-cutting solutions to multiple constraints. For instance, sorghum, millet, bambara groundnut, lentils, and groundnuts are recommended food choices under the nutritional and water limited conditions (Table 4). In this regard, they can benefit low-income producers and consumers of food who are limited in their capacity to adapt to increasing climatic risks (Vermeulen et al. 2012). Therefore, the promotion and inclusion (i.e., mainstreaming) of orphan crops could contribute towards address Sustainable Development Goals related to social and economic issues; specifically, SDGs 1, 2, 3, 8, and 15 (Mabhaudhi et al. 2016).

**Table 4:** Legume and cereal crop food choices recommended to combat nutritional and water deficit

Nutritional and health challenges	Recommended food choice		Recommended food choice under water limited conditions	
	Legume	Cereals	Legume	Cereals
Protein	White lentils^a^; soybean	Sorghum; wheat	Bambara groundnut; groundnut	Sorghum
Carbohydrates	Bambara groundnut; lentils	Equally suitable^a^	Bambara groundnut	Sorghum; millet
Energy	White lentils^a^	Equally suitable	Groundnut	Sorghum; millet
Fat	Groundnut^a^	Equally suitable	Groundnut	Sorghum; millet
Vitamin A	Common pea^a^	–		–
Micronutrients	Soybean^a^	Equally suitable	Bambara groundnut	

^a^Alternative choice due to superior nutritional content

Orphan crops can also contribute to promoting food and livelihood security and empowering vulnerable communities economically and in a sustainable manner. This is particularly important for vulnerable groups, especially women, as it has been shown to improve their socio-economic standing within their homes and communities as their families and friends have a greater respect for them (Hlahla et al. 2016). Overall, orphan crops are culturally acceptable, accessible, economically fair, and affordable; nutritionally adequate, safe and healthy; and are able to optimize natural and human resources.

### Limitations to orphan crops’ adoption and potential

Despite constituting a small share of global food systems, orphan crops have the potential to contribute toward socio-economic development of low-input–low-output farming systems. However, several challenges towards their adoption and mainstreaming must be addressed. These include, but not limited to, (1) seed systems and seed production, (2) genetic, agronomy, and eco-physiology, and (3) utilisation and marketing.

Orphan crops are mainly believed to have informal seed systems, also referred to as local, traditional, or farmer seed systems. Activities in farmer seed systems tend to be integrated and localised at the farm level, whereby the farmers themselves access seed directly from their own harvest and disseminate it through exchange and barter among friends, neighbours, and relatives; and through local markets (Venter et al. 2007). Varieties are often land or mixed races and may be heterogeneous (modified through informal breeding and use) (Sperling et al. 1993). Seed of orphan crops is often of variable quality (of different purity, and physical and physiological quality) (Wekundah 2012). In addition, their seed systems are not monitored or controlled by government policies and regulations (Sperling and McGuire 2010). Rather, local technical (indigenous) knowledge and standards, social structures and norms drive their seed systems (Sperling et al. 2013). Based on the seed scientists’ perspective, good-quality seed is a prerequisite for successful crop production. It, therefore, follows that, in the absence of a well-regulated and supported seed system, increasing the production of orphan crops, even in their agro-ecological niches, remains a challenge. In this context, it may be necessary to consider changes in the way seed for orphan crops is currently produced and distributed.

Several authors have pointed out the issues of seed dormancy, poor seed and seedling establishment, low yield, susceptibility to pests and diseases, and the presence of anti-nutritional compounds (Padulosi et al. 2004; Slabbert et al. 2004). Information on seed type, plant densities, fertiliser application rates and timing, planting dates, water requirements, weed management options, pest and disease control, and harvest techniques are required for sustainable production of orphan crops (Chivenge et al. 2015). This is especially important, considering that the efficient use of limited resources such as water and land can be enhanced through optimum agronomic practises. Combining optimum agronomic practices with improved varieties generated from breeding and biotechnology could help farmers to realise significant increases in yields of orphan crops. A synergy of crop and soil management practices is required to increase yield potential of orphan crops, especially if they are to be a source of food and nutrition security for smallholder farmers residing in sub-optimum environments. While there may be valuable lessons learnt from the existing methods, there is a need to develop specific robust methods for gathering and evaluating data that are appropriate to the realities of orphan crops’ production.

Depending on the crop and where it is cultivated, the type of market to which the crop is distributed and the means of doing so differ. There is need to recognise and develop a greater understanding of the operation of the informal markets through which orphan crops are currently sold and to develop research to support strategies that maximise profitability for farmers selling their produce. Bottlenecks to achieving this are the lack of robust and comparable empirical information concerning informal markets and the lack of workable models for maximising the value of orphan crops. This, in turn, is a function of an approach to understanding the economy which regards the informal economic as a deviation from the norm rather than being complementary to formal markets. There is limited policy that recognises the use of orphan crops as part of a strategy for sustainable food systems and climate-change adaptation, especially in marginalised communities. This acts as a disincentive to the development of distribution channels and adoption of the post-harvest handling techniques necessary to limit losses and widen the distribution of these crops.

## Conclusions

Despite the noted prospects for orphan crops under climate change, gaps in knowledge concerning orphan crops currently inhibit the capacity to protect and exploit the value of these crops within the scope of transformative adaptation. The extent to which public policy addresses these crops and their potential to contribute towards the United Nations Sustainable Development Goals is also limited but valid. Lack of research implies there is no robust, comparable, and reliable empirical information which can be used to advocate for policies on orphan crops. Indeed, as this paper suggests, there is a need to develop a clear agenda for research and development of these crops through concerted efforts involving all the stakeholders from farmers and consumers to researchers and policy makers. It is through these co-ordinated efforts that we are likely to see researchers, who are currently less inclined to work on these crops given the lack of any existing studies or workable intellectual framework for their analysis, engage meaningfully with other stakeholders to research and develop these crops as significant contributors to food and nutritional security globally.

Despite limited institutional support and an absence of research, orphan crops continue to be cultivated throughout the world. At present, farmers in the global South produce a range of both major and orphan crops; these crops and the diverse cropping systems in which they are produced contribute to the unique and rich tapestry of farming landscapes. While the absence of firm data makes accurate assessment of the role played by orphan crops difficult, limited evidence suggests that orphan crops play an important socio- economic role. In addition, they represent a rich heritage of genetic material which is of global importance. If nurtured, these genetic assets can play a critical role in building a robust, resilient, and economically vibrant agricultural sector to sustain our food and nutritional security under climate change.

### *Author contribution statement*

TM did the conceptualisation. TM and VGPC designed and developed the framework and concept note and led the writing of the manuscript. FM, SM, LN, and SH helped with the literature review. TM, VGPC, FM, and ATM contributed to the critical review and redrafting of the manuscript.
